# Maximal intended velocity enhances strength training-induced neuromuscular stimulation in older adults

**DOI:** 10.1007/s00421-022-05045-8

**Published:** 2022-09-16

**Authors:** Tiril Tøien, Thomas Malmo, Lars Espedal, Eivind Wang

**Affiliations:** 1 Faculty of Health Sciences and Social Care, Molde University College, Britvegen 2, 6410 Molde, Norway; 2 Norwegian Defence University College, Norwegian Armed Forces, Oslo, Norway; 3 Department of Østmarka, Division of Mental Health Care, St. Olavs Hospital, Trondheim University Hospital, Trondheim, Norway

**Keywords:** Rate of rise in muscle activation, Age, Efferent neural drive, EMG, Maximal strength

## Abstract

The age-related attenuation in neuromuscular function can be mitigated with strength training. Current recommendations for untrained and elderly recommend performing the strength training with a controlled movement velocity (CON). However, applying maximal intended velocity (MIV) in the concentric phase of movement may augment neuromuscular stimulation and potentially enhance training adaptations. Thus, applying rate of electromyography (EMG) rise (RER) recordings, we examined the acute early phase neuromuscular response to these two contraction types in quadriceps femoris during leg extension, along with actual movement velocity, in 12 older (76 ± 6 years) and 12 young men (23 ± 2 years). Results revealed that older adults had a lower one repetition maximum (1RM) than young (33 ± 9 kg vs. 50 ± 9 kg; *p* = 0.001) and lower actual velocity across relative intensities of ~ 10%, 30%, 50%, 70% and 90% of 1RM for CON and MIV (all *p* < 0.05). Older adults also had consistently reduced RER compared to young during both conditions (old: 1043–1810 μV; young: 1844–3015 μV; all *p* < 0.05). However, RER was higher in contractions with MIV compared to CON for both age groups, and across all intensities (98–674%, all *p* < 0.05). In conclusion, despite decreased maximal strength and attenuated neuromuscular response with advancing age, our results document an augmented neuromuscular activation when repetitions are performed with MIV in the concentric phase of movement.

## Introduction

Maximal skeletal muscle strength typically declines with 1% per year from ~ 40 years of age (Lindle et al. 1997). This is accompanied by a two–threefold larger decrease in the ability to develop force rapidly (RFD; rate of force development) (Unhjem et al. 2016; Skelton et al. 1994). The attenuation of these force-related parameters is likely due to alterations in both neural and muscular factors. Interestingly, however, is that the reduction in maximal muscle strength appears to be 3–5 times larger than the reduction in muscle mass, implying that the neural component may be the protagonist in the age-related neuromuscular attenuation (Clark et al. 2013; Bruce et al. 1997). Indeed, RFD is also recognized to be closely associated with neural factors involved in force production, particularly in the early phase (Andersen and Aagaard 2006). The age-related reductions in maximal strength and RFD have important implications for physical function and fall prevention (Wang et al. 2017), where the ability to carry out force-demanding daily tasks, such as stair walking and chair rising (Unhjem et al. 2019), or regaining postural balance to prevent falls relies heavily on RFD (Caserotti et al. 2008; Orr et al. 2006; Hester et al. 2021).

Strength training represents an excellent countermeasure to the age-related attenuation in neuromuscular function, force-related parameters, and physical function (Aagaard et al. 2010). Maximal muscle strength of older master athletes may even exceed the one observed in healthy, moderately trained, young individuals (Unhjem et al. 2016). Older adults are also shown to have a great potential for strength-training induced adaptations following short term (< 2 months) strength training interventions, with similar magnitude of improvement as observed in young (Kittilsen et al. 2021). Although both neural (Tøien et al. 2018b) and muscular (Wang et al. 2017) factors are shown to demonstrate great plasticity following strength training, it may be of particular interest to stimulate neural factors as they potentially are more prone to attenuation with advancing age. Interestingly, in light of this notion, a high intensity strength training program with emphasis on neural adaptations (maximal strength training; MST), showed that older adults regained their maximal muscle strength to a level that was similar to young after only 8 weeks (Wang et al. 2017).

Current recommendations for older adults are to perform strength training at a moderate training intensity (60–70% of one repetition maximum; 1RM) using a controlled, moderate concentric contraction velocity, lasting for approximately 2 s (Ratamess et al. 2009; Chodzko-Zajko et al. 2009). However, a faster contraction velocity may be preferable. First, functionally important RFD appears to predominantly be determined by maximal voluntary activation in the early phase of a fast muscle contraction (Maffiuletti et al. 2016), and has been linked to the rate at which motor units can be recruited (Dideriksen et al. 2020). Thus, it is conceivable that strength training focusing on fast movement velocities is more potent in effectively stimulating motor unit discharge rates during the initial phase of movement, ultimately targeting RFD. Secondly, muscle strength increases the most at the specific velocity of movement at which the training is performed (Coyle et al. 1981; Kanehisa and Miyashita 1983; Almasbakk and Hoff 1996; Coburn et al. 2006). Muscle strength during fast functional movement may be of importance for the older adults, such as in regaining postural balance to prevent falls. However, of importance, as elegantly documented by Behm and Sale (1993), it is the intended rather than the actual movement velocity that determines the velocity-specific training response. Consequently, strength training with maximal intended velocity (MIV) likely yields a similar stimulation of motor unit discharge rates as strength training with actual high velocity movement. A maximal intended, rather than actual, velocity may also be safer to perform in older and frail populations (Berg et al. 2021; Helgerud et al. 2020; Mosti et al. 2011; Cešeiko et al. 2020).

Given the potential key role of neural factors for improvements in maximal muscle strength and RFD with advancing age, exercise that aims to effectively stimulate motor unit discharge rates and target maximal voluntary activation in the early phase of a fast muscle contraction is sought after. This may be achieved through strength training with fast movement velocity, or fast intended velocity, rather than strength training with a moderate, controlled movement velocity. The primary aim of the current study was to investigate if strength training performed with MIV in the concentric phase of movement yielded superior muscle activation in the initial phase of muscle contractions over a wide range of intensities in older adults. Moreover, the secondary aim was to compare the responses to young individuals to examine if a potential difference could be impaired with age. Specifically, for both older adults and young applying intensities from ~ 10% to 90% of 1RM, we hypothesized that the application of MIV would result in higher rate of rise in muscle activation, assessed by EMG in the quadriceps, compared to the same intensity using slow-controlled movement velocity (CON).

## Methods

### Subjects

A total of 24 males participated in the study; 12 older adults (76 ± 6 years, 83 ± 16 kg, 178 ± 8 cm) were recruited from local senior citizen societies in the Trondheim, Norway and 12 young (23 ± 2 years, 74 ± 6 kg, 179 ± 6 cm) were recruited among university students and other local arenas. Participants were moderately physically active. The young group reported participation in student sports or endurance-based activities 2–3 times per week, whereas the older adults reported participation in recreational physical activities 1–3 times per week. None reported any injuries or illness that could interfere with the study. Exclusion criteria included prior competition in strength sports and a history of systematic strength training. Individuals diagnosed with cardiopulmonary diseases, musculoskeletal diseases or neurological diseases in 2 years leading up to the study were also excluded. Finally, participants were excluded if they failed to perform the entire test protocol. The data collection was carried out in accordance with the Helsinki declaration, all subjects signed informed consents, and the study was approved by a local ethical board.

### Experimental protocol

All tests were performed in the following order for each participant 1) collection of descriptive data and anthropometric measurements (thigh muscle volume), 2) unilateral maximal strength measured as one repetition maximum (1RM), and 3) measurement of movement velocity and rate of rise in muscle activity (RER) at ~ 10%, 30%, 50%, 70% and 90% of 1RM via electromyography (EMG) with different intentional velocity. All strength measurements were conducted dynamically using the dominant leg in a leg extension apparatus (IT 9005, Impulse Fitness, Taiwan), following a 5-min general warm up on an ergometer bike (Monark Ergomedic 839 E, Monark Exercise, Vansbro, Sweden).

### Anthropometry

Thigh muscle volume of the dominant foot was calculated by measuring skinfolds, length, and circumference of the thigh. Length and circumferences were measured with measuring tape. The length of the femur was measured from trochanter major to the lateral femoral epicondyle. The mid-point of the femur was defined as the length of the femur divided by two. Circumference was measured 10 cm distal and proximal to, and at the marked mid-point. Skinfold measurements were conducted utilizing the Harpenden skinfold calliper (Baty international, RH15 9LR, England) at three points around the marked mid-point of the thigh (lateral, anterior and medial). All measurements were taken 3 times at each point, and the average values were used in the analysis. Muscle volume was calculated using the following equation (Râdegran et al. 1999):

1 $${\text{Vol}}_{{{\text{anthropo}}}} = \,\,\,\left( {L/12\pi } \right) \cdot \left( {{\text{C1}}^{2} + {\text{C2}}^{2} + {\text{C}}3^{2} } \right){-}\left[ {\left( {S - 0.4} \right)/2} \right] \cdot L \cdot \left[ {\left( {{\text{C}}1 + {\text{C}}2 + {\text{C}}3} \right)/3} \right],$$

*L* refers to the length of the femur, C1, C2 and C3 is the circumference of the thigh at the distal, mid-point and proximal area. *S* refers to the skinfold thickness measurement. To account for a possible overestimation in muscle volume we utilized the equation of Layec et al. (2014), which has been validated against proton magnetic resonance imaging:

2 $${\text{Thigh muscle volume}}\left( {{\text{cm}}^{{3}} } \right) = 0.866 \cdot {\text{Vol}}_{{{\text{anthropo}}}} - 1750.$$

### Maximal strength

Maximal strength, represented by 1RM, was tested in the dominant leg, identified as the foot the participants would kick a ball with. The knee was flexed at a 90-degree angle between the leg and the horizontal plane of the muscle action. To minimize movement the subjects were strapped down at the chest and thigh and a reference point was provided to minimize head movement. Subjects were instructed to utilize the handles on the side of the leg extension apparatus. The specific warm up was performed seated in the leg extension apparatus. The subjects performed 6 sets of 8–4 repetitions with increasing submaximal load. Each warmup set was separated by a 1-min rest period. 1RM was identified as the maximum amount of weight added to the leg extension apparatus, with the subject reaching a pre-determined height of the weight stack (~ 10° knee flexion). Resistance was increased with 2–9 kg between each successful trial, and each trial was separated by a 2–3-min rest period. When the subjects were no longer able to fulfil the requirements of the test, an additional 2.5 kg was added for a last attempt, ensuring a true 1RM. All attempts were visually confirmed by a test supervisor. All subjects reached 1RM within 4–7 trials.

### Movement velocity

Following the detection of 1RM and a subsequent 3-min rest period the subjects performed a total of 10 contractions at 5 different intensities (~ 10%, 30%, 50%, 70% and 90% of 1RM) that differed in intentional velocity. In one condition, the participants were instructed to move the lever arm using a controlled, moderate contraction velocity, lasting 1–2 s, as recommended by ACSM (Ratamess et al. 2009; Chodzko-Zajko et al. 2009). This is referred to as the control condition (CON). In the other condition the participants were instructed to perform the concentric action as fast as possible, emphasizing a maximal intentional velocity (MIV), as previously used in our laboratory (Wang et al. 2017; Unhjem et al. 2021). The purpose of applying MIV is to maximally stimulate the neural descending motor drive to contracting musculature and thus to maximally recruit muscle fibers (Tøien et al. 2018b; Unhjem et al. 2021; Behm and Sale 1993). Importantly, the actual movement velocity is relatively slow, especially at higher intensities, despite the maximal intended velocity. The order of MIV and CON was randomized, while the order of intensities was standardized, with a stepwise increase from ~ 10% to 90% of 1RM. Each trial was separated by a 2-min rest period. The lightest condition was performed without external load, i.e., where the participant only had the weight of their lower leg and the lever arm of the apparatus. The weight of the foot and calf was calculated by multiplying bodyweight with 0.061, as described in Bernstein (1967). On average this weight amounted to 13% of 1RM, and as such is referred to as ~ 10% of 1RM. The duration of the concentric contraction and distance of the movement was measured through GymAware optical encoder (kinetic, Canberra, ACT) and used to calculate movement velocity.

### Muscle activation

For every trail we calculated RER, determined as the slope of the filtered EMG signal from onset of contraction to peak activation (ΔEMG/Δtime), referred to as rate of rise in muscle activation (Aagaard et al. 2002a). Peak EMG amplitude, i.e., the highest EMG amplitude recorded during the concentric phase of muscle activation, was recorded and is referred to as maximal muscle activation (Aagaard et al. 2002a). Muscle activity measurements were conducted through EMG electrodes placed on rectus femoris (RF) and vastus lateralis (VL) during 1RM and at ~ 10%, 30%, 50%, 70% and 90% of 1RM. To measure electrical activity in the muscles, six self-adhesive biopolar Ag/AgCI electrodes (Ambu, M-00-S/50, Ballerup, Denmark) were placed on RF and VL (interelectrode distance 25 mm). The electrodes were placed in accordance with the surface electromyography for the non-invasive assessment of muscle (SENIAM) recommendations (Hermens et al. 2000). Prior to attaching the electrodes, the skin was shaved, abraded (Nuprep, Weaver and company, Aurora, CO) and wiped clean with alcohol, ensuring minimal resistance between the electrodes and the skin (maximal interelectrode impedance < 5 Ω). The EMG signals were obtained using a ME6000 Biomonitor (Mega electronics, Kuopio, Finland) at 2 kHz with a common mode rejection ratio 100 dB, amplified and bandpass filtered (8–500 Hz) mediated through Megawin software 700,046 version 3.0. (Mega electronics, Kuopio, Finland). To control for potential age-related differences in conduction velocity (Aagaard 2003; Reaz et al. 2006), maximal M-wave (M_max_) was obtained in VL and RF by applying a 1 ms square-wave stimuli to the femoral nerve. The stimuli were delivered using a current stimulator (DS7AH, Digitimer, Welwyn Garden City, UK) and bipolar feltpad electrodes (8 mm in diameter, 25 mm between tips, Digitimer) at the point eliciting the highest M-wave measurements. When this point was identified, *M*_max_ amplitude during rest was determined by increasing the current amplitude by 10–40 mA, until no further increase in *M*_max_ amplitude was observed. EMG results were not normalized to *M*_max_ as there was no significant difference in *M*_max_ between older adults and young in neither RF (*p* = 0.452) or VL (*p* = 0.765).

### Statistical analysis

The statistical analysis was conducted utilizing IBM SPSS statistics 27 (Chicago, IL). Figures were constructed in GraphPad prism 8 (GraphPad software, San Diego, CA). Results are presented as means ± standard deviation (SD) in text and tables, and as mean ± standard error (SE) in figures unless otherwise stated. The significance level was set to *p* ≤ 0.05. Normal distribution was assessed by a Shapiro–Wilks test and visually through inspection of Q–Q plots. A repeated measures ANOVA was conducted to investigate the within group differences in contractions with CON and MIV. A univariate ANOVA was used to assess differences in subject characteristics, 1RM and velocity during 1RM in older adults and young. In addition, univariate ANOVA was used to assess the difference between conditions (i.e., MIV-CON) in velocity, peak EMG, and RER at ~ 10%, 30%, 50%, 70% and 90% of 1RM, between older adults and young.

## Results

### Subject anthropometry

There was no difference in height and weight between older adults and young. Age was, as expected, different between the two groups (*p* < 0.001). There was no difference between older adults and young in thigh muscle volume (old: 7.34 ± 1.58 cm^3^ vs. young: 7.89 ± 0.93 cm^3^, *p* > 0.05).

### Maximal strength and movement velocity

**Fig. 1:**
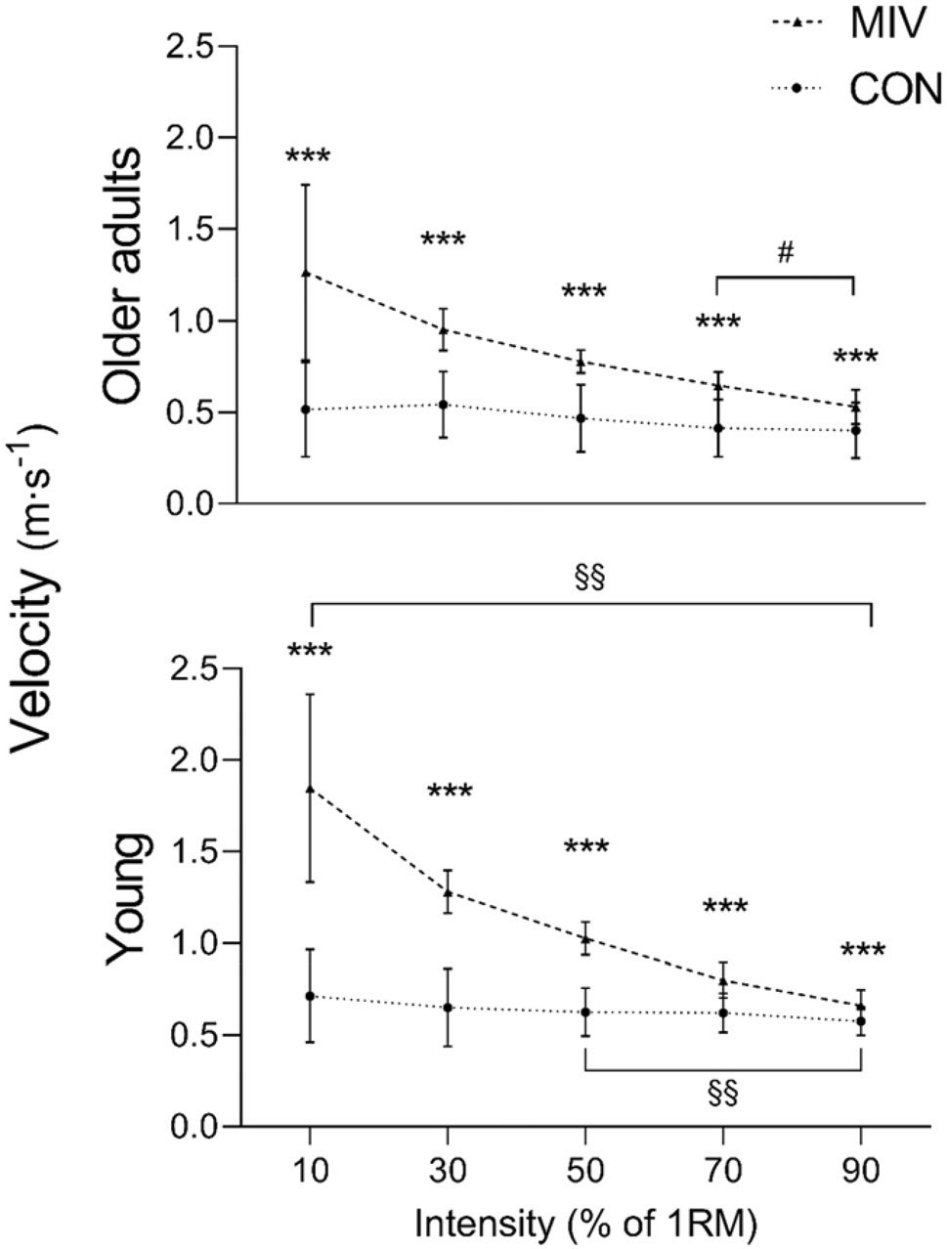
Actual movement velocity in the concentric phase at increasing intensities from ~ 10% to 90% of one repetition maximum (1RM) in older adults and young as a result of a contraction performed with maximal intended velocity (MIV) and controlled movement velocity (CON). Data are presented as mean ± SD. *MIV higher than CON; ^#^larger difference between MIV and CON compared to young; ^§^higher velocity in young compared to old. One, two, and three symbols indicate *p* ≤ 0.05, 0.01, and 0.001, respectively

1RM was lower in older adults compared to young (33 ± 9 kg vs. 50 ± 9 kg; *p* < 0.001), apparent as a 38% strength deficit. Similarly, movement velocity was higher in young compared to older adults in all contractions with MIV (*p* < 0.05), and in CON at 50%, 70% and 90% (*p* < 0.05; Fig. 1).

Velocity was consistently higher for all contractions with MIV compared to CON (all *p* < 0.001; Fig. 1). At 70% and 90% there was a larger difference in movement velocity with MIV compared to CON for older adults, when compared to young (*p* ≤ 0.05; Fig. 1).

### Rate of rise in muscle activation

**Fig. 2:**
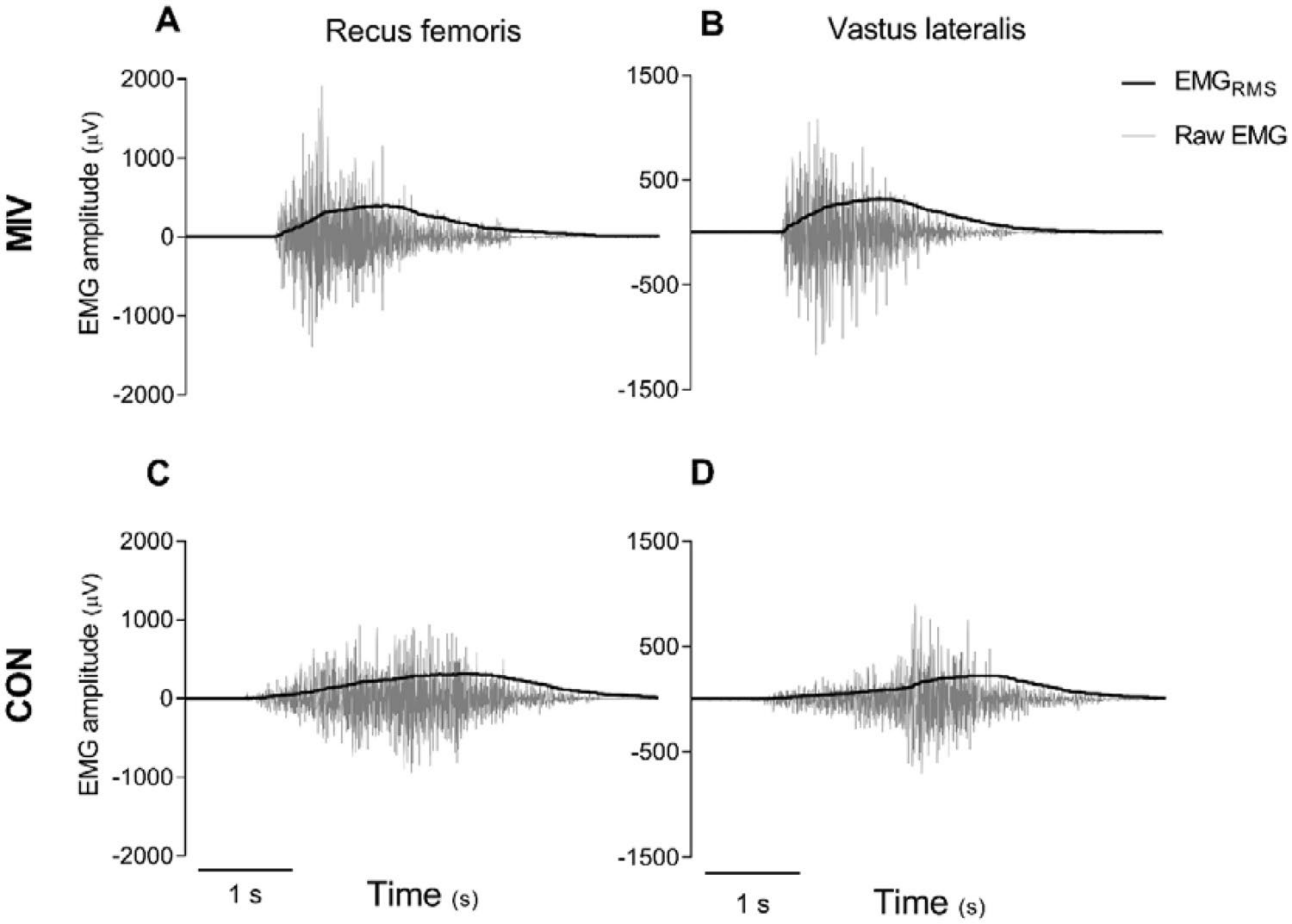
Rectus femoris (**A**, **C**) and vastus lateralis (**B**, **D**) raw electromyography (EMG) and smoothed EMG_RMS_ data from a typical young subject performing one contraction with maximal intended velocity (MIV; **A**, **B**) and controlled movement velocity (CON; **C**, **D**)

**Fig. 3:**
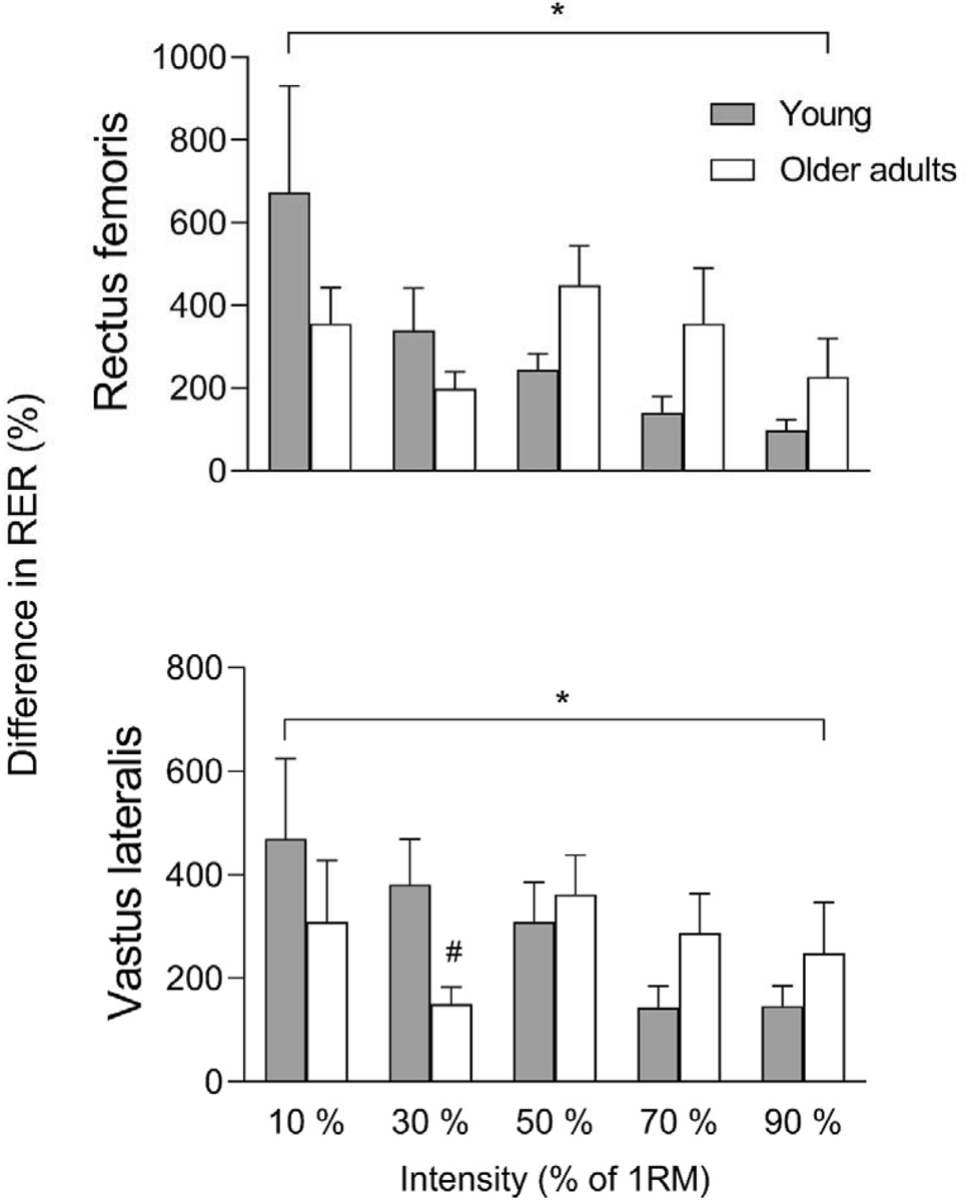
Percentage higher rate of rise in EMG (RER) during a concentric contraction performed with maximal intended velocity (MIV) than during a controlled movement velocity (CON) in rectus femoris and vastus lateralis in young and older adults. Data are presented as mean ± SE. *MIV higher than CON; ^#^smaller percentage difference between MIV and CON compared to young. One symbol indicates *p* ≤ 0.05

RER was higher in contractions with MIV compared to CON in both RF and VL in older adults and young at all intensities (all *p* < 0.001; Table 1). Young had higher RER with MIV compared to older adults at ~ 10%, 30%, and 70% of 1RM in RF and at 30% and 90% of 1RM in VL (all *p* < 0.05, Table 1). Moreover, young had higher RER compared with older adults during CON at 30%, 70% and 90% of 1RM in RF and at 70% in VL (all *p* < 0.05, Table 1). A typical response in RER during the two contractions, MIV and CON, is shown in Fig. 2. Older adults and young both displayed a similar percentage difference in RER between a contraction with and without MIV, although there was a smaller percentage difference in older adults compared to young at 30% of 1RM in VL (*p* < 0.05, Fig. 3).

**Table 1:** Rate of electromyography (EMG; µV/s) rise in muscle activation (RER) in the concentric phase of movement at ~ 10%, 30%, 50%, 70% and 90% of one repetition maximum (1RM) with maximal intended velocity (MIV) and in a controlled movement velocity (CON) in older adults and young

Intensity	Older adults (*n* = 12)		Young (*n* = 12)	
	MIV	CON	MIV	CON
Rectus femoris				
10%	1066 ± 839***	309 ± 334	2434 ± 1540***#	513 ± 425
30%	1098 ± 765***	409 ± 259	3015 ± 2707***#	786 ± 567#
50%	1652 ± 1503***	371 ± 253	2276 ± 1333***	800 ± 730
70%	1043 ± 412***	361 ± 229	2059 ± 1381***#	1051 ± 856#
90%	1184 ± 627***	573 ± 438	1846 ± 1215***	1060 ± 668#
Vastus lateralis				
10%	1271 ± 642***	426 ± 213	2048 ± 1391***	565 ± 487
30%	1125 ± 553***	527 ± 425	2418 ± 1279***##	653 ± 457
50%	1810 ± 1232***	439 ± 241	2570 ± 1817***	825 ± 682
70%	1369 ± 545***	466 ± 330	1844 ± 923***	974 ± 688#
90%	1417 ± 624***	673 ± 477	2082 ± 769***#	1070 ± 647

Data are presented as mean ± SD

*MIV higher than CON within group

^#^RER higher in young compared to older adults in the same condition. One, two, and three symbols represent *p* ≤ 0.05, 0.01, and 0.001, respectively

### Peak muscle activation

Peak EMG was higher in contractions with MIV compared to CON in older adults and young in RF in all intensities up to 50% of 1RM and up to 70% of 1RM in VL (all *p* < 0.05; Table 2). Young had higher peak EMG with MIV compared to older adults in all intensities in RF and in all intensities except ~ 10% 1RM in VL (all *p* < 0.05). Young also had higher peak EMG in contractions with CON compared to old in intensities 30–90% 1RM in RF and 50–90% 1RM in VL. Older adults and young both displayed a similar percentage difference in peak EMG between a contraction with and without MIV, although there was a smaller percentage difference in older adults compared to young at 30% of 1RM in VL (*p* < 0.05).

**Table 2:** Peak electromyography (EMG; µV) in the concentric phase of movement at ~ 10%, 30%, 50%, 70% and 90% of one repetition maximum (1RM) with maximal intended velocity (MIV) and in a controlled movement velocity (CON) in older adults and young

Intensity	Older adults (*n* = 12)		Young (*n* = 12)	
	MIV	CON	MIV	CON
Rectus femoris				
10%	242 ± 84***	162 ± 94	468 ± 276***#	304 ± 237
30%	309 ± 112*	269 ± 98	566 ± 368*#	521 ± 339#
50%	316 ± 128*	273 ± 94	620 ± 370*#	568 ± 330##
70%	319 ± 109	292 ± 113	596 ± 306##	635 ± 390##
90%	335 ± 121	306 ± 105	645 ± 396#	638 ± 411#
Vastus lateralis				
10%	336 ± 124*	269 ± 110	448 ± 294*	346 ± 295
30%	390 ± 123***	353 ± 96	607 ± 325***#	447 ± 233
50%	395 ± 112*	368 ± 71	628 ± 254*##	542 ± 272#
70%	393 ± 112*	373 ± 136	661 ± 370*#	546 ± 234#
90%	412 ± 140	405 ± 133	642 ± 338#	620 ± 272#

Data are presented as mean ± SD

*MIV higher than CON within group

^#^Peak EMG higher in young compared to older adults in the same condition. One, two, and three symbols represent *p* ≤ 0.05, 0.01, and 0.001, respectively

## Discussion

Controlled, moderate concentric contraction velocity combined with moderate intensity is commonly recommended for older individuals, who are inexperienced with strength training. However, applying MIV in the concentric phase may be more efficacious in stimulating neuromuscular function. The main finding in the present study was that contractions with MIV resulted in consistently higher rate of rise in muscle activation at all intensities between ~ 10% and 90% of 1RM contrasted to CON in both age groups. Although the response was blunted for both conditions in older adults, the difference between MIV and CON was not impaired with age. These acute effects suggest that MIV should be emphasized to target, and stimulate, neuromuscular function in the early phase of contraction, preferably in combination with high intensity to induce maximal strength improvements, particularly for older adults, where a maximal strength and RFD deficit is present.

### The influence of maximal intended velocity on rate of muscle activation

Contractions with MIV resulted in higher rate of muscle activation, represented by a higher RER, compared to contractions with CON, across all intensities and in both age groups. Thus, despite a blunted response in older adults compared to younger, a similar pattern with different intended contraction velocity was observed. Since RER is an expression of efferent neural drive in the early phase of contraction (Aagaard et al. 2002a), these acute results suggest that MIV can be incorporated to maximally stimulate motor unit discharge rate and/or motor unit recruitment, two components of efferent neural drive (Aagaard et al. 2002a, b). Moreover, ballistic contractions are shown to recruit high-threshold motor units, which are recruited last according to the recruitment hierarchy (Henneman 1957), at an earlier stage due to the instant burst of action potentials (Desmedt and Godaux 1977). Thus, contractions performed with MIV appears to enhance stimulation of neuromuscular function by enforcing high efferent neural drive to the skeletal muscle and recruitment of high threshold motor units at the early phase of contraction.

The rate of muscle activation has previously been linked to RFD (Van Cutsem et al. 1998; Aagaard et al. 2002a). In fact, the main determinant of RFD appears to be motor unit recruitment speed (Dideriksen et al. 2020). Since RFD, especially in the early phase, is a crucial physiological feature for older adults, strength training for this age group should aim to target RFD (Reid and Fielding 2012). Thus, by including MIV in strength training for older adults, which appears to primarily target motor unit discharge rate, it is likely that functional improvements will occur. In fact, previous chronic training studies, where MIV has been utilized in conjunction with high intensity (≥ 80% of 1RM), both early phase RFD and efferent neural drive has improved in both young (Tøien et al. 2018a, 2021) and older adults (Tøien et al. 2018b; Unhjem et al. 2015, 2021). In contrast, when strength training is performed without an emphasis on MIV, improvements in early phase RFD are not always observed (Andersen et al. 2010).

Although the numerical RER values (Table 1) appear to be highest at moderate intensities (~ 50% of 1RM) before levelling off at the highest intensities, it should be considered that the latter contractions were of longer duration. This increases the time spent to reach maximal activation, resulting in levelling-off or decreased RER, although peak EMG amplitude continued to increase up to the highest intensity. This is an important feature of MIV, in that it is an intentional velocity, whereas the actual velocity may be slow. This is particularly true at high intensities, where the actual movement velocity is slow, as evident from Fig. 1. However, it is the intended rather than actual velocity which appears to be important for improvements in RFD (Behm and Sale 1993), presumably resulting from elevated motor unit discharge rate along with recruitment of the fastest and strongest muscle fibres. In fact, by increasing the time under tension with maximal discharge rate when utilizing high intensity and MIV, chronic improvements in efferent neural drive has previously been documented in older adults, which was not the case following ballistic contractions (i.e., high actual velocity without external load) (Unhjem et al. 2021).

### The influence of maximal intended velocity on peak neural activation

Maximal muscle activation, represented by peak EMG amplitude, was higher in contractions with MIV at low and moderate intensities compared to CON. However, at high intensity there was no difference in maximal activation between intended contraction types. This suggests that at high intensity maximal activation is necessary to successfully overcome the external load regardless of intended contraction velocity. Moreover, since peak EMG amplitude continued to increase numerically up to the highest intensity, it suggests that maximal strength is tightly linked to maximal muscle activation, i.e., when all relevant motor units have been activated (Allen et al. 1995). Thus, for stimulation of maximal strength, the application of heavy loads appears to be more important than intentional movement velocity from the present results. Consequently, to maximize both RFD improvements and maximal strength, heavy loads along with maximal intended velocity may be advantageous.

### Movement velocity, maximal intended velocity, and age

Older adults had lower 1RM and movement velocity with MIV compared to younger adults. In fact, in most neural measurements obained in the present study, older adults displayed a blunted response compared to young. Although the difference in 1RM influences the comparison of neuromuscular response between older and younger adults, these results are in line with previous research, where efferent neural drive appears to be largely responsible for the maximal strength and RFD deficit in older adults (Unhjem et al. 2015, 2016; Clark et al. 2013). Of importance, muscular factors, such as, e.g., changes in fibre type distribution (Lexell 1995), along with decreased contractility of single fibres and dereased specific force per CSA (Larsson et al. 1997), likely also contributed to the age-related attenuation of maximal strength and RFD observed in the present study. However, again, it should be emphasized that a similar neuromuscular pattern was observed in young and older adults regarding neuromuscular response to MIV compared to CON. Thus, although the absolute training intensity must be lower in older adults, the same relative intensity and application of MIV to enhance efferent neural drive may be used in both age groups.

### Practical implications

Finding the optimal training intensity and movement velocity is important for improved physiological performance. Since RFD typically is reduced in older adults, due to decreased efferent neural drive, strength training that targets efferent neural drive in the early phase of contaction is likely important for this age group. The present study revealed that contractions with MIV enforces higher rate of muscle activation at every intensity between ~ 10% and 90% of 1RM, suggesting that the intended movement velocity is of importance for enhanced RFD. Maintaining a high RFD with older age has major implications for physical function, such as the ability to rise from a chair and climb stairs (Unhjem et al. 2019) and regain postural balance to prevent falls (Caserotti et al. 2008; Orr et al. 2006; Hester et al. 2021). Moreover, for improvements in maximal strength, our results suggest that a high intensity should be included to obtain maximal activation of motor units. We have previously shown that this combination, i.e., strength training with high intensity (80–90% 1RM) and MIV, yielded large improvements in maximal strength, RFD and efferent neural drive in older adults (Tøien et al. 2018b; Unhjem et al. 2015, 2021). It may also induce additional beneficial effects on skeletal muscle work efficency (Berg et al. 2018a, b). Moreover, ballistic contractions, i.e., MIV without an external resistance, resulted in only small improvements in maximal strength and RFD and did not affect efferent neural drive in older adults (Unhjem et al. 2021). Thus, in contrast with current guidelines (Ratamess et al. 2009), it is our recommendation that high external resistance and MIV can be used ensure improvements in physiological performance along with neuromuscular function in older and younger adults.

## Conclusions

The present study revealed that muscle contractions with MIV resulted in increased rate of muscle activation at intensities ranging from ~ 10% to 90% of 1RM compared to CON, irrespective of age. The study also revealed that maximal muscle activation was similar between intended contraction types at high intensities. These combined results suggest that strength training yields a stronger neuromuscular stimulus when it is performed with MIV compared to moderate controlled velocity, and consequently has the potential to induce greater improvements in RFD. A high training intensity may be more important for improvements in maximal strength. As such, a combination of high intensity and MIV may be preferred to maximise improvement in both these physiological characteristics when designing chronic training programs for older adults.

## Data Availability

Data are available upon reasonable request from the corresponding author.
